# Native herbivores and environmental heterogeneity as mediators of an exotic grass invasion

**DOI:** 10.1002/ece3.2727

**Published:** 2017-02-08

**Authors:** Cody L. Ender, Caroline E. Christian, J. Hall Cushman

**Affiliations:** ^1^ Department of BiologySonoma State UniversityRohnert ParkCAUSA; ^2^ Department of Environmental Studies & PlanningSonoma State UniversityRohnert ParkCAUSA

**Keywords:** drivers of invasion, environmental heterogeneity, *Holcus lanatus*, invasive exotic grass, long‐term effects, native ungulate herbivores, soil characteristics

## Abstract

Given that many exotic plant species throughout the world are having large ecological and economic effects, it is vital to understand the forces that mediate their success in novel landscapes. Both native herbivores and recipient ecosystems can have substantial effects on the performance of exotic plant species, and may interact with each other or vary in their effects over time. Unfortunately, few studies have evaluated the importance of these kinds of context‐dependent effects. Here, we use a 17‐year‐old exclosure experiment stratified across a coastal grassland in northern California to address the relative importance of a reintroduced mammalian herbivore, tule elk (*Cervus canadensis nannodes*), and environmental heterogeneity in mediating the growth, abundance, and recruitment of a problematic grass invader, *Holcus lanatus*. We found that elk reduced *Holcus* abundance, aboveground biomass, percent cover, frequency, and seedling recruitment, but that these effects often varied among habitat types, with effects being greater in open grasslands than shrub‐dominated grasslands. The performance of *Holcus* populations also varied significantly among habitat types, with the invader usually having the greatest success in *Baccharis*‐dominated grasslands. Our results suggest that environmental heterogeneity had much greater influence on *Holcus* success than elk, and that these effects were due largely to soil pH and moisture. The negative effects of elk on *Holcus* appeared after 4 years and did not intensify after an additional 13 years. Furthermore, despite their negative effects, these prominent herbivores did not prevent the spread of *Holcus* into previously uninvaded areas. Our research highlights the importance of assessing the individual and interactive effects of native herbivores and environmental heterogeneity on the success of invasive, exotic plant species. It emphasizes the reality that the negative effects of herbivores on exotic plant species will often vary across heterogeneous landscapes and may be insufficient to prevent the expansion of these invaders.

## 1 Introduction

Given that many exotic plant species throughout the world are having large ecological and economic effects, it is vital to understand the forces that mediate the success of invaders in their recipient landscapes. A wide variety of hypotheses have been proposed to explain the success of exotic species including enemy release (Agrawal et al., 2005; Inderjit & Cahill, 2015; Keane & Crawley, 2002), biotic resistance (Levine, Adler, & Yelenik, 2004; Parker, Burkepile, & Hay, 2006; Pearson, Potter, & Maron, 2012), invader life history traits (Corbin & D'Antonio, 2010; Rejmánek & Richardson, 1996), and resource availability (Colautti, Grigorovich, & MacIsaac, 2006; Davis, Grime, & Thompson, 2000; Koerner et al., 2015). These biotic and abiotic drivers of invasion may operate simultaneously in the same system and may interact with each other in important ways, shedding light on the forces controlling the success of invaders (Catford, Jansson, & Nilsson, 2009).

Herbivores are an important biotic characteristic of recipient communities that can influence dominant plant invaders through their activities as consumers, disturbance agents, dispersers, and fertilizers (Maron & Vila, 2001; Vavra, Parks, & Wisdom, 2007). Given their potential to impact exotic plant species, herbivores could be useful in managing invasive populations. For example, domestic livestock have been increasingly used to restore degraded grasslands dominated by exotic annual grasses (Skaer, Graydon, & Cushman, 2013; Stahlheber & D'Antonio, 2013). Once extirpated, reintroduced herbivores also have the potential to be effective tools for managing plant invasions (Johnson & Cushman, 2007; Polak & Saltz, 2011). However, for both domestic and native herbivores, studies have reported widely varying results of their impacts on exotic plant populations. Herbivores can promote (Dávalos, Nuzzo, & Blossey, 2015; Kalisz, Spigler, & Horvitz, 2014; Knight, Dunn, Smith, Davis, & Kalisz, 2009; Relva, Nunez, & Simberloff, 2010; Vavra et al., 2007), deter (Case & Crawley, 2000; Cushman, Lortie, & Christian, 2011; Eckberg, Tenhumberg, & Louda, 2014; Parker et al., 2006; Pearson et al., 2012), or have no effect on the dominance of exotic plant species (Stohlgren, Schell, & Vanden Heuvel, 1999). Predicting the conditions under which herbivores suppress versus promote invasion is critical not only for effective use in restoration, but also for the study of biological invasions in general.

Differences in abiotic and biotic characteristics of recipient ecosystems may drive much of the observed variation in the success of exotic plants. By themselves, abiotic conditions of a given environment can have strong influences on the success of plant invaders (Davis et al., 2000; Weiher & Keddy, 1995). In addition, positive and negative interactions with members of the recipient community can further mediate the success and spread of exotic plant species (Badano, Villarroel, Bustamante, Marquet, & Cavieres, 2007; Cushman et al., 2011; Maron & Connors, 1996). Both biotic and abiotic characteristics can exhibit tremendous spatial heterogeneity, which is likely to mediate the effects of herbivores on invaders (Biswas, Kotanen, Kambo, & Wagner, 2014; Cushman et al., 2011; Maron & Vila, 2001). However, few studies have previously explored the degree to which effects of herbivores on plant invaders vary across heterogeneous landscapes (but see Biswas et al., 2014; Stohlgren et al., 1999). Thus, to improve our ability to generalize about the importance of herbivores and recipient environments in mediating the distribution of invasive species, it is imperative to examine the effects of herbivores along environmental gradients and in multiple habitat types.

Plant invasions have a strong temporal dimension and the effects of herbivores and recipient communities on them are also likely to vary over time (Wilson et al., 2007). For example, there are often time lags between the introduction of an exotic taxa and the species becoming invasive (Aikio, Duncan, & Hulme, 2010; Larkin, 2012; Pyšek, Hulme, & Republic, 2005). Climatic variability can also play a role in the spread of invasive plants and interact with the effects of other drivers (Cabra‐Rivas, Saldaña, Castro‐Díez, & Gallien, 2016; Taylor & Kumar, 2016). Due to this variation, the effects of herbivores and recipient environments on exotic plants may increase, decrease, or change direction over time (Britton‐Simmons & Abbott, 2008; Kalisz et al., 2014; Takagi & Miyashita, 2015; Tang, Gao, Wang, Zhao, & Li, 2012). In addition, the impacts of herbivores on plant invaders may arise soon after introduction or may become apparent (or intensify) only after some amount of time has passed. Thus, incorporating a temporal dimension has the potential to forward our understanding of how herbivores and recipient environments influence invasion processes.

In this study, we used a 17‐year‐old experiment stratified across a heterogeneous landscape to examine the effects of tule elk (*Cervus canadensis nannodes*), a reintroduced native herbivore, on an invasive exotic perennial grass, *Holcus lanatus,* along the coast of northern California. Our research addressed the following three questions: (1) What is the relative importance of native herbivores and recipient environments in mediating the growth, abundance, and recruitment of a dominant exotic grass? (2) Can the effects of a heterogeneous landscape on invader success be explained by abiotic soil differences? (3) Has the *Holcus* invasion changed over time and have herbivores, environmental heterogeneity, or their interaction mediated this trajectory? Answers to these questions will forward our general understanding of the drivers of plant invasions and will help guide more effective efforts to control invasive perennial grasses in altered landscapes. Through their activities as herbivores and disturbance agents, we predict that elk will have negative effects on the growth and abundance of *Holcus*, but positive effects on recruitment by increasing the availability of safe sites. We further predict that elk will slow *Holcus* invasion but that their influences will be highly context dependent, being greater in more accessible open grasslands than in shrub‐dominated landscapes where elk might be deterred by dense shrub growth.

### 1.1 Study system

Our research was conducted on Tomales Point in Point Reyes National Seashore, approximately 65 km northwest of San Francisco. Bordered by the Pacific Ocean and Tomales Bay, Tomales Point is a 1,030‐ha peninsula that experiences a Mediterranean‐type climate, with moderate rainy winters and cool, foggy summers with very little precipitation. The coastal grasslands on Tomales Point consist of both native and exotic herbaceous plant species interspersed with native shrubs. Three distinct habitat types occur within our 300‐ha study area: *Baccharis*‐dominated grasslands, *Lupinus*‐dominated grasslands, and open grasslands. Open grasslands occur on the Kehoe soil formation (derived from Cretaceous granitic parent rock; Kashiwagi, 1985) and are dominated by herbaceous species and largely devoid of shrubs (Johnson & Cushman, 2007). *Baccharis*‐dominated grasslands occur on a subvariant of the Kehoe formation (Kashiwagi, 1985) and are characterized by herbaceous patches mixed with dense stands of *Baccharis pilularis* (Asteraceae), a long‐lived native shrub (Johnson & Cushman, 2007). *Lupinus*‐dominated grasslands are located on a mix of soil formations, either completely in Sirdrak sand (derived from a Quaternary dune sandstone parent rock) or a mixture of Sirdrak sand and Kehoe variant (Kashiwagi, 1985). The latter soils are extremely well‐drained, resulting in much drier conditions in the *Lupinus*‐dominated grasslands than in *Baccharis*‐dominated or open grasslands (V. Dodge and J. H. Cushman, *unpublished data*). *Lupinus*‐dominated grasslands are predominantly open areas interspersed with a short‐lived, native, nitrogen‐fixing shrub, *Lupinus arboreus* (Fabaceae).

Tule elk (*C. canadensis nannodes*) is a native ungulate that previously dominated much of coastal and central California. These herbivores once numbered 500,000 individuals, but hunting and land conversion during the Gold Rush brought them to the brink of extinction by the mid‐1800s (McCullough, 1969). The dramatic decline prompted efforts to protect elk, bolster their numbers, and reintroduce populations to over 20 different sites in California. In 1978, 10 tule elk were reintroduced to a designated wilderness area on Tomales Point. Following their reintroduction, the tule elk population grew rapidly for two decades, reaching approximately 450 individuals before leveling off. Since 1998, the herd has typically fluctuated between 400 and 600 individuals, although censuses between 2014 and 2016 indicated that the population has declined to fewer than 300 animals, possibly due to prolonged drought (D. Press, *unpublished data*). The diet of tule elk at Tomales Point consists primarily of herbaceous forbs and grasses, but they also consume shrub foliage during the winter months when there is less herbaceous material available (Gogan & Barrett, 1995).

The exotic perennial grass *H. lanatus* (velvet grass; hereafter referred to as *Holcus*) is originally native to Eurasia but has been introduced widely throughout the world. It is particularly problematic and widespread throughout coastal regions of Australia and the United States, where it was likely introduced as forage seed either intentionally or as a contaminant (Thompson & Turkington, 1988). The earliest record of *Holcus* in California is from San Francisco in 1886 and herbaria records show that this grass was found on the Point Reyes Peninsula by 1898 (data provided by the participants of the Consortium of California Herbaria; ucjeps.berkeley.edu/consortium/). This grass grows best in moist conditions, but is able to withstand moderate periods of drought and is more susceptible to trampling than most pasture plants (Thompson & Turkington, 1988). *Holcus* has become a problematic and widespread invader in California and the California Invasive Plant Council has designated it as having substantial ecological impacts due to its ability to form dense monocultures that reduce species richness of native grasses and forbs (Cal‐IPC, 2010; Corbin & D'Antonio, 2010; Deck, Muir, & Strauss, 2013).

## 2 Methods

### 2.1 Herbivore‐exclosure experiment

This study centers around a large‐scale elk exclosure experiment located on Tomales Point in Point Reyes National Seashore. Established by the National Park Service and US Geological Survey in 1998, the experiment occurs within a 300‐ha area and consists of 24 36 × 36 m plots distributed equally among three habitat types—*Baccharis*‐dominated, *Lupinus*‐dominated, and open grasslands. Each plot in the experiment is located 350–850 m from the Pacific Ocean. Within each of the three habitat types, there are four pairs of plots, with one plot within each pair randomly assigned fencing to exclude elk and another plot spaced 3 m away left unfenced to serve as a control. The fencing that surrounds each exclosure plot is 2.5‐m tall and effectively excludes elk, but not other small‐ or mid‐sized herbivores such as deer or hares (J. H. Cushman, *personal observation*). Other studies using this exclosure experiment have shown that elk exert major influences on the plant community (Johnson & Cushman, 2007; Lee, Spasojevic & Cushman, *unpublished data*; Richter, Spasojevic & Cushman*, in review*), small mammals (Ellis & Cushman, *in review*), ground‐dwelling arthropods (Cecil & Cushman, *unpublished data*), plant functional traits (Lee, Spasojevic & Cushman, *unpublished data*), and soil characteristics (Dodge, Eviner & Cushman, *unpublished data*).

### 2.2 *Holcus* responses

To assess the effects of elk on an exotic perennial grass, we quantified growth, abundance, and recruitment responses of *Holcus* in the exclosure experiment in late May of 2015, just before this grass started to senesce. We stratified 12 50 × 50 cm quadrats within each of the 24 plots in the exclosure experiment, uniformly spacing them in a 4 × 3 grid. We restricted sampling to the center 30 × 30 m of each plot to reduce edge effects and sampled only in areas without shrub cover, relocating quadrats landing beneath shrub canopies to the nearest open space in order to remain consistent with methods used by Johnson and Cushman (2007). In each of the 12 quadrats per plot, we quantified the percent cover, frequency, and abundance of *Holcus*. We assessed percent cover using standard point‐intercept sampling at 16 points within each quadrat and frequency within 25 cells of each quadrat. Due to the clonal nature of *Holcus*, it was often difficult to distinguish individual plants. Following Johnson and Cushman (2007), when estimating plant abundance, we defined an individual as a clump of culms and tillers (young vegetative shoots) unattached to other clumps by stolons. We also assessed frequency and abundance of *Holcus* seedlings and juveniles—small, non‐flowering plants with no branched culms—in order to estimate successful recruitment from seed.

We quantified aboveground biomass using five 25 × 25 cm quadrats stratified within each plot—in the center of each plot and in the center of each plot quarter. We clipped all non‐woody plants at ground level, separated *Holcus* biomass from other plant matter, and then dried the biomass for 48 hr at 60°C prior to weighing.

To assess individual plant responses, we quantified height, inflorescences per adult, and specific leaf area (SLA; leaf area/dry mass). We measured maximum height of *Holcus* and the number of inflorescences in each of the 12 previously described 50 × 50 cm quadrats per plot. In early June of 2015, we harvested 10 fully formed, undamaged *Holcus* leaves from each plot in order to quantify SLA, which is positively related to a number of measures of plant performance, including photosynthetic rate, leaf nitrogen, and relative growth rate, and negatively related to leaf longevity and secondary compounds such as lignin (Pérez‐Harguindeguy et al., 2013). We kept leaves cool and moist during transportation from the field to the lab and allowed leaves to rehydrate in wet paper towels before processing. In the lab, we scanned rehydrated leaves and measured area of the leaf lamina, excluding the leaf sheath from scanned the plant material using Image‐J software. After weighing dried leaves (dried at 60°C for 48 hr), we calculated SLA as leaf area per unit dry mass (cm^2^/g).

### 2.3 Dung deposition

To generate an estimate of elk activity, we determined the amount of dung deposited in each of the 12 control plots of the exclosure experiment in nine surveys conducted between January and May 2015. Each survey consisted of a whole‐plot count and quantified the length and width of each dung pile. The area of an ellipse was used to estimate the area of each dung pile (in our system, dung counts and dung area were highly correlated). As pointed out by Riginos and Grace (2008), Young, Palmer, and Gadd (2005), and others, dung counts are a reliable method for estimating the level of activity of mammalian herbivores within their habitats.

### 2.4 Soil pH and moisture

We quantified average soil pH and gravimetric moisture in each of the 24 plots in the exclosure experiment to test whether these variables predicted the success of *Holcus*. Between February 2015 and April 2016, we quantified soil pH at nine locations on a 3 × 3 grid in each plot using a Kelway pH meter (Kelway Products, Wyckoff, NJ, USA). In March 2015, we collected soil samples from these same nine locations stratified within each plot to assess soil gravimetric moisture. We used a soil corer to collect samples from the top 10 cm of soil and weighed the soil before and after drying for 72 hr at 60°C. We quantified soil moisture as mass of oven‐dried soil divided by mass of field‐wet soil (g/g).

### 2.5 Statistical analyses

We analyzed each of the *Holcus* response variables using linear mixed models in JMP 12 (SAS Institute, Cary, NC, USA), with elk (present or excluded), habitat type (*Baccharis*‐dominated, *Lupinus*‐dominated, and open grasslands) and their interaction as fixed effects and plot pair (1–12) nested within habitat type as a random effect. For all response variables except SLA, we nested quadrat within plot pair and treated it as a random effect. We used the Kenward–Roger method (Kenward & Roger, 1997) to estimate error degrees of freedom, which is widely recognized as the most rigorous approach when using linear mixed models (Bolker et al., 2009; Kenward & Roger, 1997; Schaalje, McBride, & Fellingham, 2002). For SLA, we used plot‐level averages in our statistical analyses. To ensure that assumptions for linear mixed models were met, we visually assessed all model residuals for approximate normality and checked for homoscedasticity of residual plots. Total abundance and seedling abundance were square‐root‐transformed and seedling frequency was square‐root‐log‐transformed to meet normality assumptions. If habitat type or any interaction terms were significant in our models, we followed up with Tukey multiple comparison tests to evaluate differences among the means. As outlined in Edwards, Muller, Wolfinger, Qaqish, and Schabenberger (2008), we calculated the relative effect size of all fixed factors in mixed models as marginal *R* ^2^ values (=[no. of fixed effects × *F*/*df* Den]/[1 + no. of fixed effects × *F*/*df* Den], where *F* is the *F* ratio statistic, and *df* Den is denominator degrees of freedom). These values describe the proportion of variance explained by a fixed factor on its own.

In addition to assessing the influence of elk on *Holcus* variables using a categorical predictor variable (herbivores present vs. excluded), we have evaluated whether varying *levels* of elk activity, as estimated by dung cover in the control plots of the exclosure experiment, predicted the *magnitude* of change in *Holcus* abundance, biomass, or cover due to elk. For each of the 12 plot pairs (control and exclosure plots), we quantified the magnitude of the elk effect on *Holcus* using log‐response ratios (LRR: ln[*Holcus* value in control plot/*Holcus* value in exclosure plot]). We then analyzed LRR values for *Holcus* abundance, biomass, cover, and frequency using ANCOVAs, with dung area, habitat type, and their interaction as fixed effects.

We used multiple regression analysis to determine the degree to which soil pH and moisture predicted *Holcus* abundance, independent of elk. The regression consisted of soil pH and gravimetric moisture as independent variables and *Holcus* abundance as the dependent variable. We log‐transformed *Holcus* abundance to meet normality assumptions.

We tested whether the presence of *Holcus* in each of the 24 plots varied by year using contingency table analysis. We originally used habitat type, year (2002 or 2015), and *Holcus* presence (yes or no) as the focal variables, but due to absolute presence or absence of *Holcus* in some habitat types in some years, this analysis resulted in unstable *F* and *p* values. Instead, we performed separate analyses for *Lupinus*‐dominated and open grasslands to look for variation among habitat types with year and *Holcus* presence as the focal variables. Because the *Baccharis*‐dominated grassland was a perfect predictor of *Holcus* (100% presence in 2002 and 2015), we did not include this habitat type in the contingency analysis.

We also evaluated the effects of elk on the invader over time using data collected by Johnson and Cushman (2007), whose methods were comparable to the ones described in this study. We calculated the elk effect on *Holcus* abundance and aboveground biomass as the LRR (=ln [elk present/elk excluded]). We analyzed the *Holcus* response to elk in a mixed model with year (2002/2003 or 2015) and habitat type as fixed effects and pair nested within habitat type as a random effect.

## 3 Results

**Figure 1:**
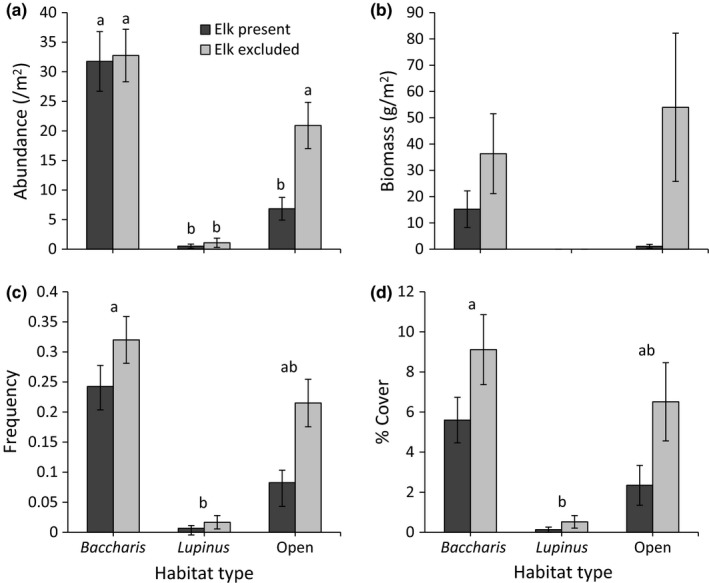
Mean (±1*SE*) abundance (a), aboveground biomass (b), frequency (c), and percent cover (d) of *Holcus lanatus* as a function of elk (present or excluded) and habitat type (*Baccharis*‐dominated, *Lupinus*‐dominated, or open grassland). Letters above bars correspond to the results from Tukey multiple comparison tests. Although *Holcus* was present in some of the *Lupinus*‐dominated grassland plots, our biomass quadrats did not capture those individuals, resulting in zero *Holcus* biomass

**Figure 2:**
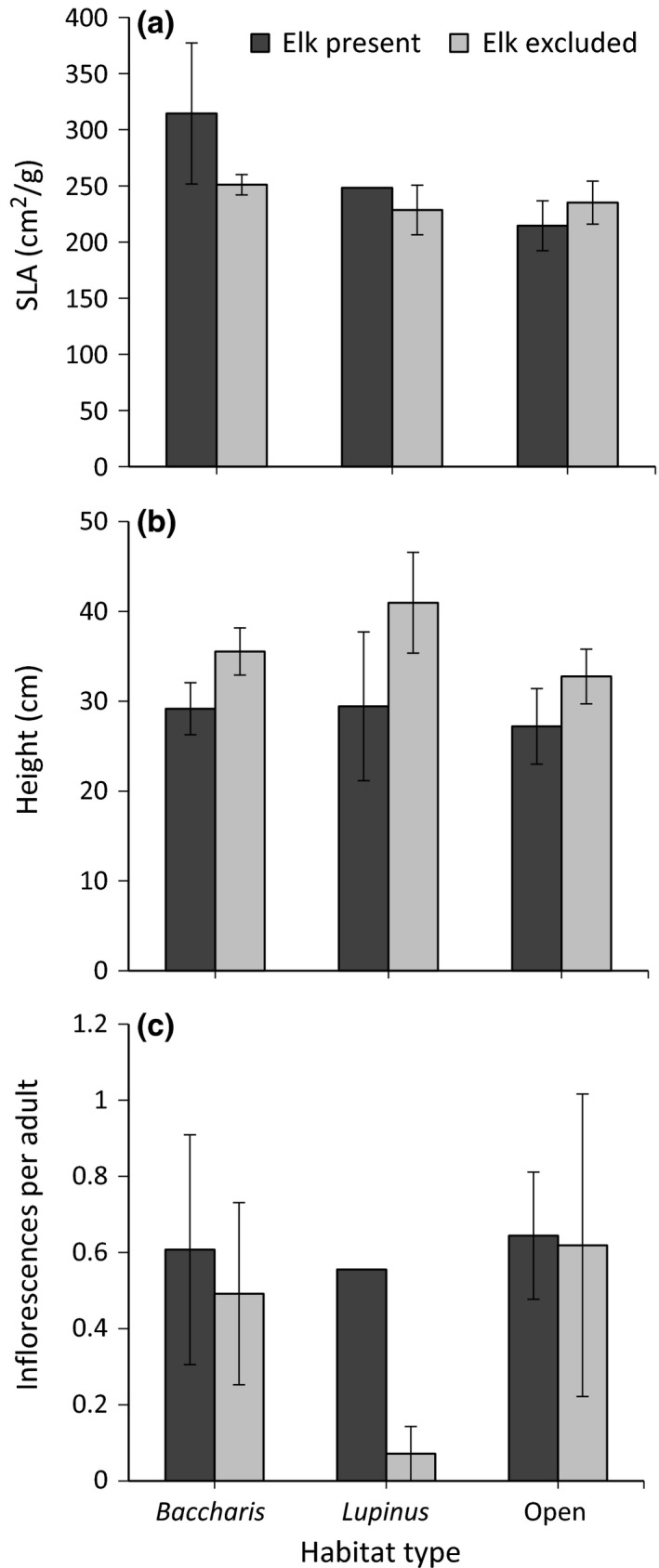
Mean (±1*SE*) specific leaf area (SLA) (a), plant height (b), and average number of inflorescences per adult individual, (c) of *Holcus lanatus* as a function of elk (present or excluded) and habitat type (*Baccharis*‐dominated, *Lupinus*‐dominated, or open grassland). Three of four of the control plots in *Lupinus*‐dominated grasslands contained no *Holcus*, so bars for SLA and inflorescences per adult represent the plot‐wide averages of the one remaining plot and lack error bars

Our results document that elk significantly reduced *Holcus* abundance, aboveground dry biomass, frequency, and percent cover (Figure 1, Table 1a–d). In addition, abundance, frequency, and percent cover—but not aboveground biomass—varied significantly among habitat types, with values being greatest in *Baccharis*‐dominated grasslands, least in *Lupinus*‐dominated ones, and intermediate in open grasslands (Figure 1, Table 1a–d). The effect of elk on *Holcus* abundance varied significantly among habitat types, with similar trends for biomass and frequency (Figure 1a–c, Table 1a–c). In each case, the effect of elk was greatest in open grasslands and weak or absent in *Baccharis*‐ and *Lupinus*‐dominated grasslands. In contrast to these patterns, the effect of elk on percent cover did not vary among habitat types (Figure 1d, Table 1d). We failed to detect an effect of elk, habitat type, or their interaction on *Holcus* SLA or mean inflorescences per adult (Figure 2a,c, Table 1e and g). We detected a trend for elk to decrease plant height, but neither habitat type nor the elk × habitat interaction had an influence (Figure 2b, Table 1f). In all models, the marginal *R* ^2^ value for significant effects was largest for habitat type (*R* ^2^ = .72–.90), intermediate for elk (*R* ^2^ = .14–.20), and smallest for the elk × habitat interaction (*R* ^2^ = .06; Table 1).

**Table 1:** Results from linear mixed models evaluating the effects of tule elk and habitat type on *Holcus* (a) abundance (square‐root‐transformed), (b) aboveground biomass, (c) frequency, (d) percent cover, (e) seedling abundance (square‐root‐transformed), (f) seedling frequency (square‐root‐log‐transformed), (g) specific leaf area (SLA), (h) height, (i) average inflorescences per adult, (j) elk effect on abundance, and (k) elk effect on aboveground biomass

Response	Fixed effect	*df*	*F*	*p*	Marginal *R* ^2^
(a) Abundance (sqrt)	Elk	1, 141	8.85	.0034	.16
	Habitat type	2, 9	26.28	.0002	.90
	E × HT	1, 141	3.17	.0448	.06
(b) Biomass	Elk	1, 57.5	4.83	.032	.20
	Habitat type	2, 9.1	1.57	.2591	–
	E × HT	2, 57.5	1.96	.1495	–
(c) Frequency	Elk	1, 141	10.50	.0015	.18
	Habitat type	2, 9	10.78	.0041	.78
	E × HT	2, 141	2.45	.09	–
(d) Percent cover	Elk	1, 141	7.76	.0061	.14
	Habitat type	2, 9	7.76	.011	.72
	E × HT	2, 141	1.45	.2369	–
(e) SLA	Elk	1, 10.8	0.33	.5759	–
	Habitat type	2, 9.2	1.62	.2497	–
	E × HT	2, 9.0	0.71	.5175	–
(f) Height	Elk	1, 45.2	2.28	.1377	–
	Habitat type	2, 11.5	0.32	.7321	–
	E × HT	2, 63.8	0.09	.9119	–
(g) Inflorescences per adult	Elk	1, 42.3	0.56	.4571	–
	Habitat type	2, 11.4	0.26	.7778	–
	E × HT	2, 58.0	0.07	.9307	–
(h) Seedling abundance (sqrt)	Elk	1, 141	3.46	.065	–
	Habitat type	2, 9	23.90	.0003	.89
	E × HT	1, 141	4.14	.018	.08
(i) Seedling frequency (sqrt ln)	Elk	1, 141	4.95	.0277	.10
	Habitat type	2, 9	30.04	.0001	.91
	E × HT	1, 141	2.82	.0628	–
(j) Elk effect on abundance	Year	1, 9	0.01	.9098	–
	Habitat type	2, 9	0.24	.7948	–
	Y × HT	2, 9	0.33	.7261	–
(k) Elk effect on biomass	Year	1, 9	0.34	.5718	–
	Habitat type	2, 9	4.18	.052	–
	Y × HT	2, 9	2.51	.1359	–

**Figure 3:**
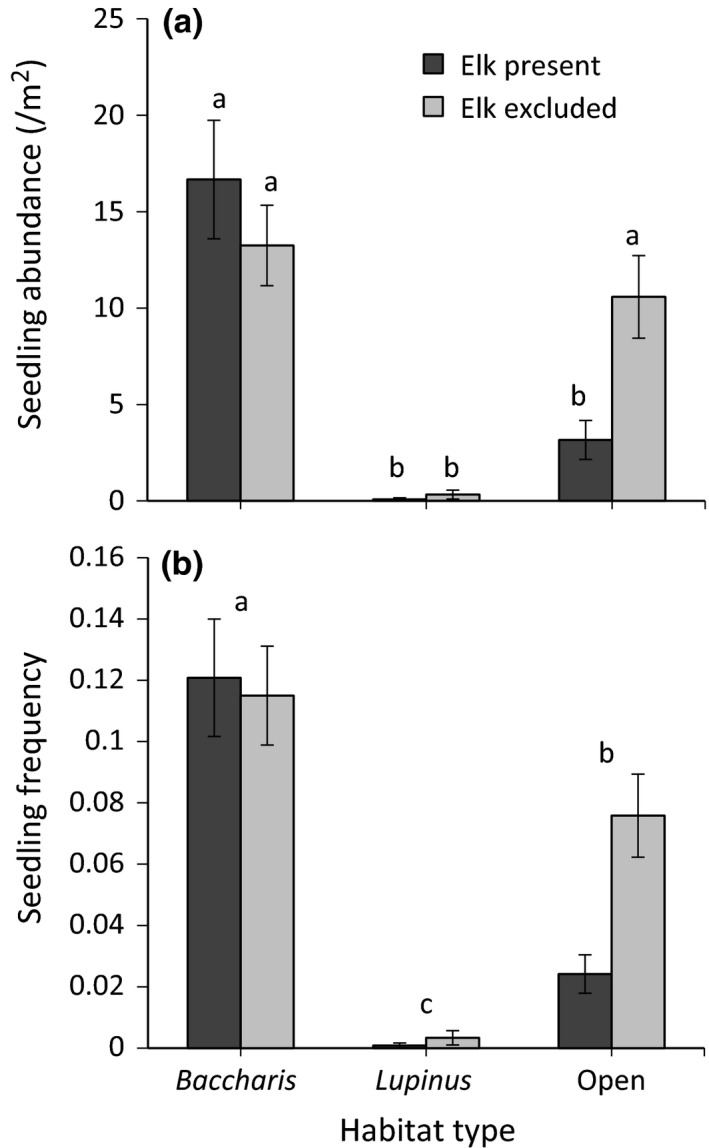
Mean (±1*SE*) seedling abundance (a) and seedling frequency (b) of *Holcus lanatus* as a function of elk (present or excluded) and habitat type (*Baccharis*‐dominated, *Lupinus*‐dominated, or open grassland). Letters above bars correspond to the results from Tukey multiple comparison tests

We detected a trend for elk to reduce the abundance of *Holcus* seedlings and this effect varied significantly among habitat types, with differences arising only in open grasslands (Figure 3a, Table 1h). Seedling abundance varied significantly among habitat types, with levels highest in *Baccharis*‐dominated grasslands, lowest in *Lupinus*‐dominated ones, and intermediate in open grasslands (Figure 3a, Table 1h). Elk also significantly decreased seedling frequency and values varied among habitat type, with the highest frequencies found in *Baccharis*‐dominated grasslands, followed by open grasslands, and then *Lupinus*‐dominated grasslands (Figure 3b, Table 1i). We also detected a trend for the effect of elk to vary among habitats, following the same pattern as seen for seedling abundance (Figure 3b, Table 1i). Again, the marginal *R* ^2^ values for both seedling abundance and frequency were greater for habitat type than elk or the elk × habitat interaction (Table 1h and i).

Despite the significant effect of elk treated as a categorical variable (presence, absence) on many *Holcus* response variables, we failed to detect an effect of dung cover (an estimate of elk activity *level*) on the magnitude of change in *Holcus* abundance, biomass, cover, or frequency, as assessed by LRRs (*F*_1,6_ = 0.42, *p* = .5416; *F*_1,6_ = 3.56, *p* = .1080; *F*_1,6_ = 0.69, *p* = .439; *F*_1,6_ = 0.05, *p* = .828, respectively). We also failed to detect an interaction between dung activity × habitat type on the magnitude of change in *Holcus* variables (abundance, *F*_2,6_ = 0.06, *p* = .9385; biomass, *F*_2,6_ = 1.13, *p* = .382; cover, *F*_2,6_ = 0.34, *p* = .726; frequency, *F*_2,6_ = 0.23, *p* = .80).

**Figure 4:**
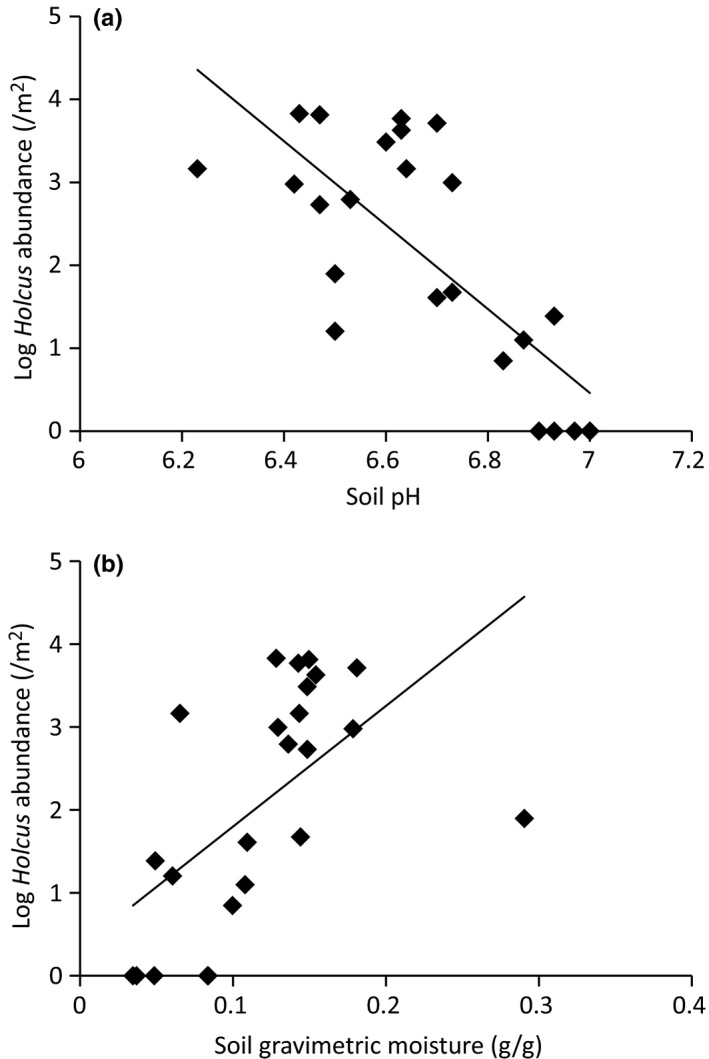
The log‐transformed abundance of *Holcus lanatus* per plot as a function of soil pH (a) and soil moisture (b)

*Holcus* abundance decreased significantly with increasing soil alkalinity (Figure 4a; *F*_1,21_ = 12.04, *p* = .0023) and increased significantly with increasing soil moisture (Figure 4b; *F*_1,21_ = 5.68, *p* = .0266). Overall, these two effects accounted for 58% of the variation in *Holcus* abundance (*F*_2,21_ = 14.71, *p* = .0001).

**Figure 5:**
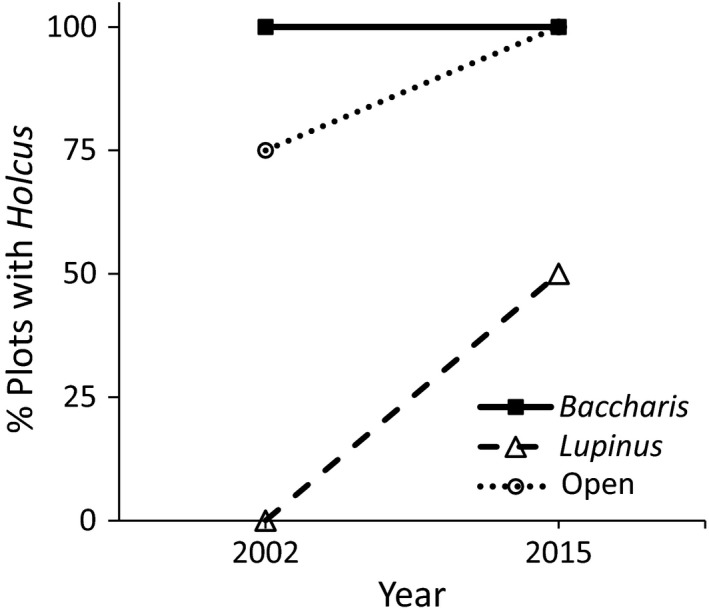
Percent of plots containing *Holcus lanatus* in 2002 and 2015 across three habitat types (*Baccharis*‐dominated, *Lupinus*‐dominated, and open grassland)

**Figure 6:**
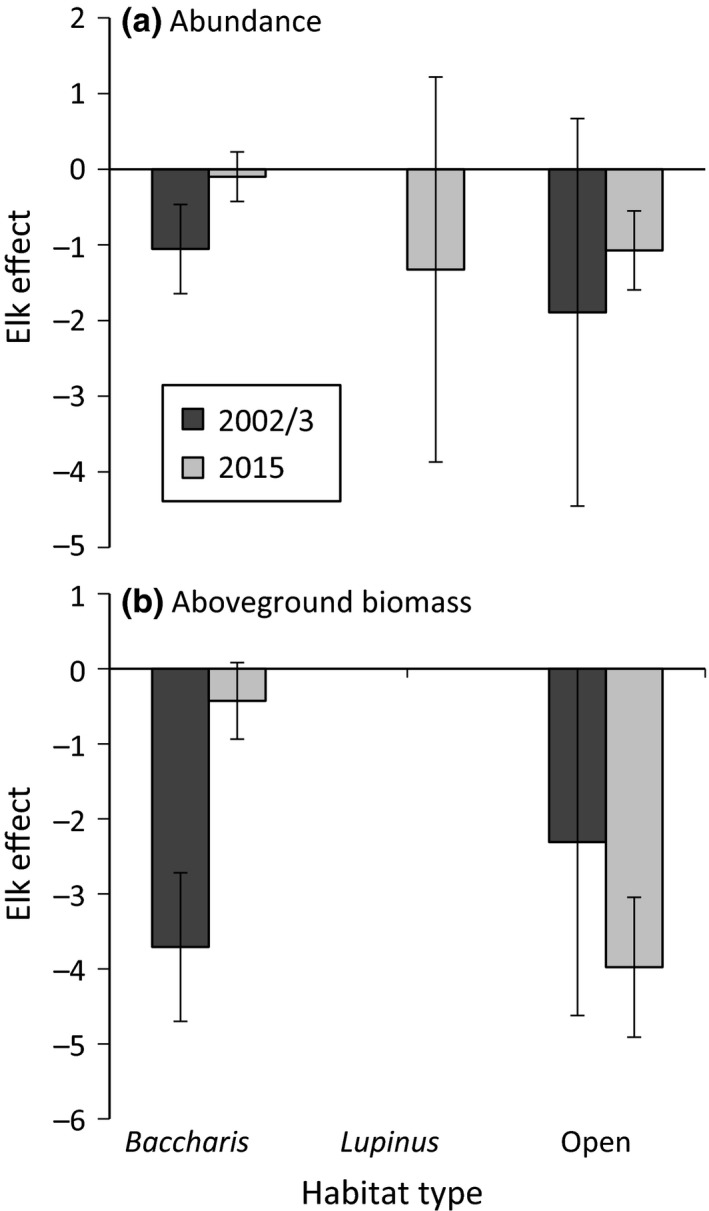
Mean (±1*SE*) elk effect (log response ratio = ln [elk present/elk excluded) ]on *Holcus lanatus* abundance (a) and aboveground biomass (b) as a function of year (2002/2003 or 2015) and habitat type (*Baccharis*‐dominated, *Lupinus*‐dominated, or open grassland)

*Holcus* was present in all of the *Baccharis*‐dominated grassland plots in 2002 as well as 2015, increased from 75% to 100% in the open grassland plots and increased from 0% to 50% of the plots in *Lupinus*‐dominated grasslands (Figure 5). We detected a trend for the presence of *Holcus* to vary between years in open grasslands (LR χ^2^ = 3.06, *df* = 1, *p* = .0803) and this relationship was significant in *Lupinus*‐dominated grasslands (LR χ^2^ = 6.90, *df* = 1, *p* = .0086). Interestingly, the elk effect on *Holcus* abundance and aboveground biomass did not vary between the two sample years but did vary among habitat types, with there being negative effects in *Baccharis*‐dominated and open grasslands but no effect in *Lupinus*‐dominated grasslands (Figure 6, Table 1j and k). However, we did detect a trend for the effect of elk on *Holcus* biomass to decrease over time in *Baccharis*‐dominated grasslands, while it remained constant in *Lupinus*‐dominated and open grasslands (Figure 6b, Table 1k). The elk effect on *Holcus* abundance did not vary neither among habitat types nor with the interaction of habitat and year (Figure 6a, Table 1j).

## 4 Discussion

Using a 17‐year‐old exclosure experiment, we have shown that a reintroduced native herbivore and environmental heterogeneity both play important roles in affecting the dominance of an extremely invasive exotic grass. Our findings demonstrate that elk negatively influenced *Holcus* populations, decreasing their local distribution, abundance, percent cover, aboveground biomass, and seedling recruitment. We also found that the population parameters we measured commonly varied substantially among different habitat types and that the effects of elk on *Holcus* varied among them as well. Soil moisture and pH explained much of the variation in *Holcus* abundance and these variables were important factors driving the heterogeneity among the different habitat types. The effect of elk on *Holcus* in 2015 was no different than that detected 13 years earlier. The relative effect size of habitat type was much greater than that for elk, and this may explain why the negative impacts of elk were not sufficient to prevent the expansion of this dominant invader into the more favorable habitat types found in our system during the past 13 years.

The enemy release hypothesis (ERH, sensu Darwin, 1859; Elton, 1958) predicts that exotic taxa should thrive in their new ranges because they are freed from control by native predators, pathogens, and herbivores. Although not a direct test of ERH, our findings that native herbivores can reduce the success of exotic plant populations joins a growing number of studies that fail to support this hypothesis. In agreement with our findings, many other studies have reported that native herbivores reduce the success of exotic plant populations (Case & Crawley, 2000; Colautti, Ricciardi, Grigorovich, & MacIsaac, 2004; Cushman et al., 2011; Keane & Crawley, 2002; Parker et al., 2006).

Although elk exerted an overall negative effect on *Holcus* populations in our study, this influence often varied among habitat types (Figure 1 and 3, Table 1). Elk typically had strong negative effects on *Holcus* in open grasslands and absent or weak effects in both *Baccharis*‐ and *Lupinus*‐dominated grasslands. We do not think that these habitat‐specific results are explained by spatial variation in the level of elk activity in our exclosure experiment. This is because we found that the amount of dung deposited by elk in plots—an estimate of their activity level—did not predict the magnitude of the elk effect on *Holcus*. Rather, we hypothesize that the effects of elk were minimal in *Baccharis*‐dominated grasslands because the dense shrub cover of this habitat type protected the invader from herbivores (Johnson & Cushman, 2007). Alternatively, the invasion in *Baccharis*‐dominated grasslands may have reached its full spatial extent at the time the experiment was established. Elk may be more effective at slowing the spread of the invasion rather than reducing *Holcus* in plots already heavily invaded.

We hypothesize that the negative effects of elk on *Holcus* populations were caused by the combined effects of herbivory and disturbance, resulting in greater mortality of seedlings and juvenile plants and thus decreased recruitment. In support of this hypothesis, previous studies at our field site have reported that, although not a preferred food plant, elk will consume *Holcus,* with the species constituting up to 12% of its diet during the summer months (Gogan & Barrett, 1995; Roberts, 2000). In New Zealand and England, both Crawford and Liddle (1977) and Edmond (1964) also report that *Holcus* was reduced by livestock and human trampling, and was more sensitive to these disturbances than other grass species. At our site, elk cause substantial disturbance to the soil and vegetation (J. H. Cushman, *unpublished data*; Johnson & Cushman, 2007) and we hypothesize that this trampling is a major factor explaining the negative effects of elk on *Holcus* populations.

A lack of effect on individual responses but a decrease in *Holcus* abundance and seedling recruitment leads us to hypothesize that elk reduced the survival of young plants but had little negative effects on established individuals. Since elk did not affect the number of inflorescences per plant, and presumably seed production, the reduced number of seedlings can be attributed to a reduction in safe sites, due to either reduced germination rates or increased seedling mortality. Although we did not quantify germination rates, we hypothesized that ground disturbance and reduced competition due to elk activity would increase favorable germination sites in control plots. Our seedling abundance data did not support this hypothesis. In contrast, increased mortality of delicate young plants due to trampling and/or consumption could explain the negative effects of elk on seedling recruitment. While few studies have quantified the effects of large native herbivores on exotic plant recruitment, our results agree with other studies showing that various smaller native herbivores and granivores suppressed exotic seedling recruitment. For example, Case and Crawley (2000) found that rabbits reduced seedling recruitment and survival of an invasive exotic forb in Great Britain. Additionally, in a review of 18 studies, Maron and Vila (2001) found that native herbivores decreased seedling performance (seed set, seed viability, and seedling recruitment) by a third.

The magnitude of the elk effect on *Holcus* biomass and abundance did not change with increasing duration of the exclosure experiment, except for a trend to decrease in *Baccharis*‐dominated grasslands when measured by aboveground biomass. Although we do not know if or how the elk effect varied in the intervening years, the negative effects of elk on *Holcus* were evident 4 years into the experiment—if not earlier—and were still evident and not significantly different 13 years later. As Levine et al. (2004) and Maron and Vila (2001) point out, generalist herbivores may have negative effects on exotic plant species, but it is unclear whether they can prevent invasive establishment or eradicate already established exotic populations. Our results provide support for this skepticism, since the negative influence of elk did not increase over time and was not enough to prevent the advance of *Holcus* into previously uncolonized plots. We believe this occurred because, although elk negatively affect recruitment of new individuals, they likely do not completely eliminate them. Furthermore, we hypothesize that elk have minimal effects on older, established plants. Thus, we suspect that any *Holcus* seedlings that are able to survive will persist, if not thrive, in elk grazed landscapes. In contrast to our study, Pearson et al. (2012) found that granivorous rodents reduced percent cover and reproduction of an exotic aster (*Tragopogon dubius*) and provided biotic resistance to the community by severely limiting local abundance of this invader. Additionally, the effect of small mammals increased over time as populations of *T. dubius* grew within exclosures. The study by Pearson et al. (2012) diverges from ours in that it excluded granivores, who may preferentially prey upon the seeds of certain species, thus having stronger and more specific effects than the generalist herbivores in our experiment.

In addition to the effects of elk, we found that environmental heterogeneity had a major influence on *Holcus* abundance, cover, frequency, and seedling recruitment (Figures 1 and 2). We consistently observed that *Holcus* populations were least successful in *Lupinus*‐dominated grasslands, most successful in *Baccharis*‐dominated grasslands, and intermediate in open grasslands. Our data suggest that much of this variation in *Holcus* performance was due in part to soil differences among the three habitat types. The *Lupinus*‐dominated grasslands have a significantly higher proportion of coarse sand, lower soil moisture, and higher soil pH than the other two habitat types (Dodge, Eviner & Cushman, *unpublished data*). As shown in Figure 4, we found that *Holcus* abundance increased with soil moisture and acidity, which was also described by Thompson and Turkington (1988). These two factors accounted for over half of the variation in *Holcus* abundance and these unfavorable abiotic conditions in the *Lupinus*‐dominated grasslands have probably been the primary factor slowing the spread of *Holcus*.

In our system, it appears that the environmental heterogeneity found among the different habitat types was a greater driver of *Holcus* invasion than tule elk. In estimating the relative effect size of our fixed effects, all significant habitat effects accounted for at least 72% of the variation in *Holcus* response, whereas elk only accounted for up to 20% (Table 1). We suspect that the larger influence of habitat type explains why elk alone were not sufficient to slow the spread of *Holcus* in this system. In a long‐term, large‐scale exclosure study in a Rocky Mountain grassland, Stohlgren et al. (1999) also found that differences in climate and soil characteristics had greater effects on exotic plant species richness and cover than grazing by native and domestic herbivores. However, variables such as precipitation and many soil characteristics are beyond the control of land managers, whereas grazing can be manipulated and used as a tool to manage invasive plant species. Understanding the interaction between grazing and environmental factors will help in assessing the potential for herbivores to control exotic plant species. For example, we found that elk were effective at reducing *Holcus* cover in the open grasslands, but less effective in the other two habitat types. Thus, although environmental factors may play the largest role in determining the overall dynamics of the *Holcus* invasion, the smaller but very real effects of native herbivores on exotic invasive plant species can still be useful in making management decisions, given that these are the factors that managers can manipulate.

In conclusion, our study demonstrates that both native herbivores and environmental heterogeneity can be important drivers of exotic plant invasions and can interact with each other to mediate the success of a dominant exotic plant species. In our system, habitat type was the stronger driver of invasive plant success and mediated the effects of elk, highlighting the need to assess habitat suitability as well as biotic interactions when attempting to understand and manage the dynamics of invasive plant populations. Furthermore, our results show that the negative effects of native herbivores on exotic plant populations may not transfer into long‐term control or prevention of invasion. Thus, it is critical to study interactions between native herbivores and exotic plants across a heterogeneous landscape and over longer time periods, which will allow for greater insight about the importance and dynamics of context‐dependent outcomes in invaded systems.

## Conflict of Interest

None declared.
